# Improving Fast Charging-Discharging Performances of Ni-Rich LiNi_0.8_Co_0.1_Mn_0.1_O_2_ Cathode Material by Electronic Conductor LaNiO_3_ Crystallites

**DOI:** 10.3390/ma15010396

**Published:** 2022-01-05

**Authors:** Tongxin Li, Donglin Li, Qingbo Zhang, Jianhang Gao, Long Zhang, Xiaojiu Liu

**Affiliations:** New Energy Materials and Devices Laboratory, School of Materials Science and Engineering, Chang’an University, Xi’an 710064, China; 2018031001@chd.edu.cn (T.L.); 2019131013@chd.edu.cn (Q.Z.); 2019231009@chd.edu.cn (J.G.); 2020131022@chd.edu.cn (L.Z.); 2021231096@chd.edu.cn (X.L.)

**Keywords:** Li-ion batteries, fast charging, LiNi_0.8_Co_0.1_Mn_0.1_O_2_, LaNiO_3_, electron transport, rate capability

## Abstract

Fast charging-discharging is one of the important requirements for next-generation high-energy Li-ion batteries, nevertheless, electrons transport in the active oxide materials is limited. Thus, carbon coating of active materials is a common method to supply the routes for electron transport, but it is difficult to synthesize the oxide-carbon composite for LiNiO_2_-based materials which need to be calcined in an oxygen-rich atmosphere. In this work, LiNi_0.8_Co_0.1_Mn_0.1_O_2_ (NCM811) coated with electronic conductor LaNiO_3_ (LNO) crystallites is demonstrated for the first time as fast charging-discharging and high energy cathodes for Li-ion batteries. The LaNiO_3_ succeeds in providing an exceptional fast charging-discharging behavior and initial coulombic efficiency in comparison with pristine NCM811. Consequently, the NCM811@3LNO electrode presents a higher capacity at 0.1 C (approximately 246 mAh g^−1^) and a significantly improved high rate performance (a discharge specific capacity of 130.62 mAh g^−1^ at 10 C), twice that of pristine NCM811. Additionally, cycling stability is also improved for the composite material. This work provides a new possibility of active oxide cathodes for high energy/power Li-ion batteries by electronic conductor LaNiO_3_ coating.

## 1. Introduction

Li-ion batteries (LIBs) have received increasing attention for electric vehicles (EVs) and portable electronics due to their high energy density and long lifespan [1,2,3]. As a result, the development of LIBs with high capacity, high power/energy density, as well as long cycle life is necessary. The power density and energy density are significant parameters for LIBs [4]. Except for improving energy density, novel technologies and materials are needed to resolve the requirement for high power densities by enabling rapid charging-discharging rates without sacrificing cycling stability and energy densities. In particular, power density is critical for most applications, such as power grid stabilization and fast-charging EVs. Recently, despite the charging technology of LIBs having been intensively investigated, the current charging capability is still far from offering consumers the same refueling experience as conventional vehicles [5]. This is a significant reason, causing “range anxiety” for EVs owners and potential customers. Consequently, a higher charging rate with a shorter charging time is essential to achieve fast charging in the future [6,7,8].

Based on previous reports, multiple properties of the applied cathode, anode, and electrolyte materials affect the fast-charging capability of LIBs. Fast charging technology mainly depends on the transport rate of electrons and Li^+^ between the LIBs components. To improve the fast-charging capability of LIBs, numerous research have been devoted toward reducing the diffusion length of Li^+^ and electrons by nanotechnology (such as nanofibers, nanotubes, or nanoparticles), and increasing the electronic and ionic conductivity by hybrid-composite design, Li-ion diffusion control, surface modification, and dopant manipulation [9,10]. Previous reports have demonstrated that electronic or ionic conductors can improve electronic or ionic conductivity, providing a positive impact on the fast charging of LIBs [11].

Compared with conductive carbon anode materials, the study on the influence of fast charging on the cathodes is still in its infancy [12]. At present, developing cathodes with high capacity, high operating voltage, and long lifespan is of great importance for practical application in LIBs. Traditional cathodes, such as LiCoO_2_, LiFePO_4_, and LiMn_2_O_4_, have limited reversible capacity (<200 mAh g^−1^), which cannot satisfy the requirement for high power and energy density LIBs [13,14]. Among numerous cathodes, LiNi_0.8_Co_0.1_Mn_0.1_O_2_ (NCM811) is considered as one of the most promising cathodes due to its high specific capacity (>200 mAh g^−1^), low cost, and high average redox potential [15,16]. Nevertheless, the inferior rate performance of NCM811 could be ascribed to the Li^+^/Ni^2+^ cation mixing, caused by the similar ionic radius between Ni^2+^ (0.69 Å) and Li^+^ (0.76 Å), resulting in reduced electrode kinetics and specific capacity [17,18,19]. Additionally, the poor cycling performance is closely related to moisture sensitivity and detrimental side reactions [20,21]. More importantly, the intrinsic poor electronic conductivity (10^−5^ S cm^−1^) of NCM811 restricts its transport kinetics (e.g., rate performance) and cycling stability [22]. Moreover, these problems will worsen at high current density. To resolve the above problems, surface modification is considered as an efficient strategy to enhance the electrochemical performances of NCM811 cathodes. Among various coating materials, the majority of the lithium ionic conductors (Li_3_PO_4_ [23], Li_2_ZrO_3_ [24], Li_3_VO_4_ [25]) are focused on increasing the ionic conductivity of Ni-rich cathodes.

Furthermore, previous reports have demonstrated that electronic conductivity plays a crucial role in initiating the electrochemical process, and electron transport is critical to improving the electrode kinetics that dominate the power density of LIBs [26]. As a consequence, it is necessary to enhance the electronic conductivity of NCM811 cathode. It is well known that the high electronic conductivity carbon is beneficial to supply the electron transport pathway in the manufacturing process of LIBs. Thus, it is essential to add conductive agent (carbon black) into the conventional electrode materials. Moreover, many studies have reported that effective improvement of the electronic conductivity for NCM811 cathodes could be achieved by coating the carbon or conductive polymer. More recently, Sim et al. [27] prepared carbon-modified LiNi_0.8_Co_0.1_Mn_0.1_O_2_ cathodes using carbon black (Super P) as the carbon source, resulting in superior electrochemical performances. Furthermore, Zha et al. reported an efficient method to decorate the surface of LiNi_0.8_Co_0.1_Mn_0.1_O_2_ (NCM811) by combining with polyimide and carbon nanotubes. Compared with the modified-NCM811 (199.6 mAh g^−1^), NCM811 exhibits a higher initial discharge capacity (201.1 mAh g^−1^). However, it can be found that surface modification with carbon and conductive polymers is usually realized under an inert atmosphere and high temperature, leading to a poor rate capability or a loss in capacity. To improve the rate performance and specific capacity, it is necessary to achieve the coating process under an oxygen or air atmosphere, and the oxides could satisfy this demand.

The perovskite oxide LaNiO_3_ (LNO) has been intensively investigated in various applications in ferroelectric devices due to its highly electronic properties [28]. The rhombohedral structure of LaNiO_3_ oxide is metallic at all temperatures, and its high electronic conductivity (over 100 S cm^−1^) enough to act as an electrode [29,30,31,32,33]. Furthermore, LaNiO_3_ has been reported as a novel anode for LIBs, which exhibits superior electrochemical performances. More importantly, LaNiO_3_ could be annealed under an oxygen atmosphere. In this paper, we report the electronic conductor LaNiO_3_ as a coating layer to decorate the NCM811 surface for fast charging-discharging LIBs. The conductive LaNiO_3_ coating provides an effective electron transport pathway and serves as a protective layer that restrains the interfacial side reactions between the electrolyte and the NCM811 surface. Additionally, the impact of the LaNiO_3_ coating on the NCM811 cathodes is studied in detail.

## 2. Materials and Methods

### 2.1. Synthesis of NCM811 Cathode Materials

NCM811 was prepared via a typical sol-gel method as follows. First of all, stoichiometric amounts of Co(COOCH_3_)_2_·4H_2_O, Ni(COOCH_3_)_2_·4H_2_O, Mn(COOCH_3_)_2_·4H_2_O, and LiNO_3_ (with 5% excess) were dissolved together in ethyl alcohol to obtain a uniform solution. An excessive amount of LiNO_3_ was employed to compensate for possible lithium loss at high temperature. Afterward, acetylacetone (the molar ratio of transition metal ions to acetylacetone was 1:1) was added into the metal solution. The mixed solution was then evaporated with stirring in an 80 °C water bath. The obtained gel was dried at 100 °C and annealed at 450 °C for 2 h. After that, the acquired powder was ground, and calcined at 800 °C for 12 h in oxygen (denoted as pristine NCM811).

### 2.2. Synthesis of LaNiO_3_ Surface-Modified NCM811

A simple wet chemical method was employed to prepare the LaNiO_3_-modified NCM811 cathodes. Firstly, deionized water was used to dissolve Ni(NO_3_)_2_·6H_2_O and La(NO_3_)_3_·6H_2_O with the stoichiometric ratio of 1:1. Citric acid was then added under vigorous stirring to obtain the LaNiO_3_ transparent solution. Subsequently, the as-synthesized NCM811 sample was added into a required amount of LNO solution (from 0, 1, to 3 wt%), and the obtained suspension was stirred continuously at 80 °C to evaporate the water. Finally, the powder was dried at 100 °C and subsequently calcined at 700 °C for 3 h under flowing O_2_ to obtain the LaNiO_3_ surface-modified NCM811 materials. Based on the ratio of LNO to NCM811, the LNO-modified NCM811 materials were labeled as pristine NCM811, NCM811@LNO, and NCM811@3LNO, respectively.

### 2.3. Material Characterization

Powder X-ray diffraction (XRD, Bruker D8 ADVANCE) using a Cu target under 40 mA and 40 kV was used to characterize the crystal structure of samples. The XRD patterns were collected over the 2θ range of 15–90° with a step size of 0.02°, and the scanning rate was 2.4° min^−1^. Furthermore, Rietveld refinement program—General Structure Analysis System (GSAS) software was used to further analyze the XRD data. Scanning electron microscopy (SEM, Hitachi S-4800) was employed to characterize the morphology. Elemental distribution on the surface of samples was analyzed by energy dispersive X-ray spectroscopy (EDS).

### 2.4. Electrochemical Measurements

Furthermore, 20 wt% carbon black, 10 wt% polyvinylidene fluoride (PVDF), and 70 wt% active material were dissolved together in N-methyl pyrrolidone (NMP), forming a slurry to prepare the electrodes. The slurry was cast on Al foil and dried at 100 °C. The mass loading of the active material was approximately 1–2 mg cm^−2^. The as-prepared electrode as the working electrode, Li metal as the reference electrode, and microporous polypropylene membrane (Celgard 2500) as the separator, assembling the CR2025-type coin cells in an argon gas-filled glove box. The electrolyte was 1 M LiPF_6_ dissolved in dimethyl carbonate (DMC) and ethylene carbonate (EC) (1:1 vol/vol). After aging, a multichannel battery testing system (Neware Technology Co., Ltd., Shenzhen, China) was used to measure the electrochemical performances between 2.8 and 4.3 V. Cyclic voltammetry (CV) and electrochemical impedance spectroscopy (EIS) tests were carried out on a Princeton Applied Research VersaSTAT 3 electrochemical workstation. The EIS tests were conducted between 100 kHz and 10 mHz, and the voltage amplitude was 5 mV. The cyclic voltammetry was tested in the potential range between 2.8 and 4.5 V, and the scan rate was 0.1 mV s^−1^. The differential capacity versus voltage curves (dQ dV^−1^) were obtained according to the charge/discharge testing data of individual cycles.

## 3. Results

### 3.1. Physical Characterizations

**Figure 1:**
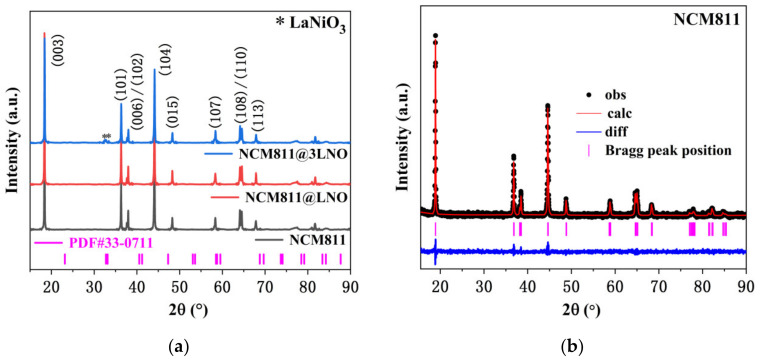

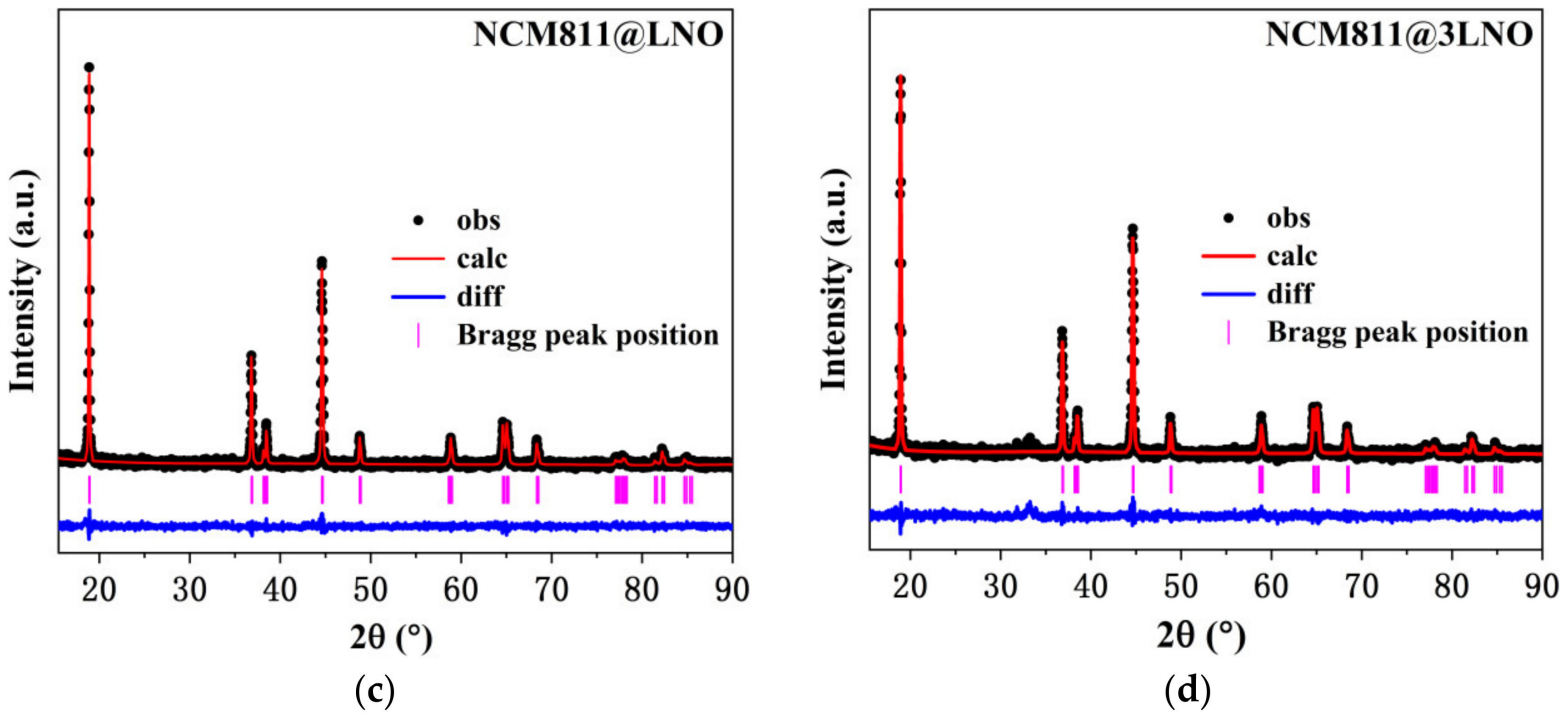
(**a**) XRD patterns of all three samples; the Rietveld refinement of (**b**) pristine NCM811, (**c**) NCM811@LNO, and (**d**) NCM811@3LNO samples.

The crystal structure of pristine NCM811, NCM811@LNO, and NCM811@3LNO samples were investigated by powder X-ray diffraction (Figure 1a) combined with Rietveld refinement (Figure 1b–d). The main diffraction peaks of NCM811@LNO and NCM811@3LNO samples are similar to those of pristine NCM811, which belong to hexagonal layered α-NaFeO_2_ structure (R−3m space group), suggesting that the LNO coating does not affect the crystal structure of NCM811 [34]. Additionally, all three samples exhibit clear splitting of (108)/(110) and (006)/(102), which implies a well-ordered layered structure [35]. Compared with pristine NCM811, NCM811@LNO and NCM811@3LNO samples exhibit a negligible change in lattice parameters (Table 1), suggesting that La^3+^ is not doped into the bulk structure. The (003)/(104) peak intensity ratio is closely related to the degree of Li^+^/Ni^2+^ cation mixing in the Li layer according to the literature [36,37]. Interestingly, NCM811@LNO and NCM811@3LNO samples exhibit higher I_(003)_/I_(104)_ values compared to the pristine NCM811, suggesting that the LaNiO_3_-modified LiNi_0.8_Co_0.1_Mn_0.1_O_2_ samples have lower Li^+^/Ni^2+^ disordering. Previous research results demonstrated that Li/Ni disordering is harmful to the kinetic diffusion of Li ion during electrochemical cycling, thus deteriorating the rate capability and discharge capacity [38]. Obviously, in the NCM811@LNO sample, no diffraction peak corresponding to the LaNiO_3_ can be seen, which may be caused by the low content of LNO material. In addition, the relatively weaker diffraction peaks between 30 and 35° could be identified as the crystalline LaNiO_3_ (JCPDS #33−0711, labeled with *) in the NCM811@3LNO sample, suggesting the successful introduction of LaNiO_3_ nanocrystals to the NCM811 [39]. Based on these results, we can speculate that the NCM811@LNO sample contains crystalline LNO owing to the identical preparation method.

**Figure 2:**
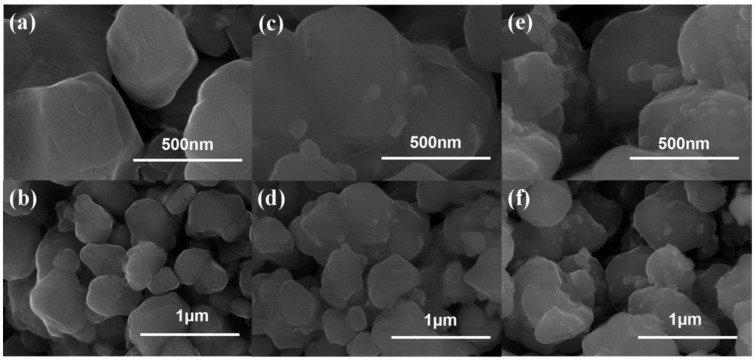
SEM images of (**a**,**b**) pristine NCM811, (**c**,**d**) NCM811@LNO, and (**e**,**f**) NCM811@3LNO samples.

**Figure 3:**
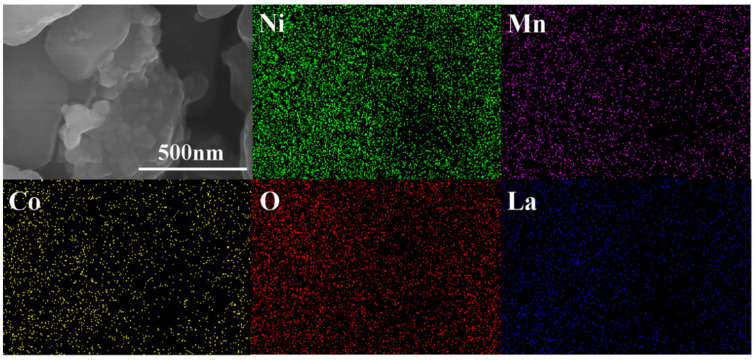
EDS elemental mapping of NCM811@3LNO sample.

All three samples are composed of well crystalline particles, and the average particle size is approximately 300–700 nm. Clearly, the pristine NCM811 exhibits smooth and clear particle surfaces (Figure 2a,b), whereas some small nanoparticles can be seen on the rougher surface of NCM811@LNO (Figure 2c,d) and NCM811@3LNO (Figure 2e,f) samples. Clearly, with increasing LNO coating content, the amount of small nanoparticles increases gradually on the NCM811 particles surface. Additionally, Figure 3 presents the EDS elemental mapping of the NCM811@3LNO sample. Obviously, Mn, Ni, Co, O, and La elements are homogeneous distribution on the particle’s surface. In addition, the atomic percentages (at.%) of La, Ni, Mn, and Co are 1.51, 38.68, 4.62, and 4.71 at.%, respectively. According to these results, it is concluded that the LaNiO_3_ can be evenly coated on the NCM811 surface by a simple wet chemical process, forming a conductive coating layer. As a consequence, the electronic conductor LNO crystallites coating can facilitate the electron transport on the surface, restrain the direct contact between the electrolyte and the cathode surface, and thus reduce the transition metal ions dissolution and the interfacial side reactions.

### 3.2. Electrochemical Performance

**Figure 4:**
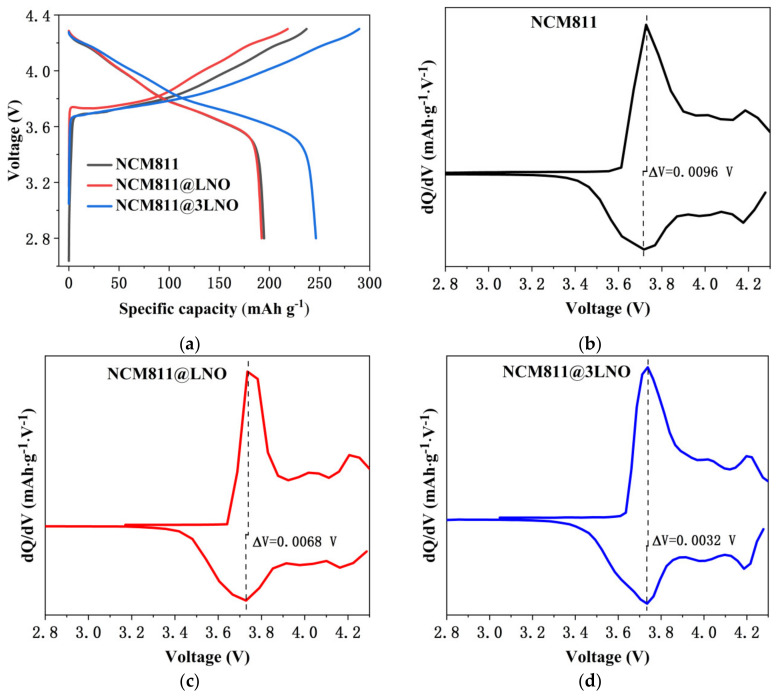
(**a**) First charge and discharge voltage profiles, and corresponding dQ dV^−1^ curves of (**b**) pristine NCM811, (**c**) NCM811@LNO, and (**d**) NCM811@3LNO electrodes at 0.1 C rate.

The first charge/discharge voltage profiles of pristine NCM811, NCM811@LNO, and NCM811@3LNO electrodes at 0.1 C (1 C = 180 mA g^−1^) are presented in Figure 4a. Compared with the charge and discharge profiles of pristine NCM811, NCM811@LNO and NCM811@3LNO electrodes do not show any additional voltage plateau. Table 2 shows the first coulombic efficiencies and charge/discharge specific capacities of all three electrodes. It is conspicuous that the NCM811@3LNO electrode exhibits an excellent coulombic efficiency of 85.10% and an ultrahigh first discharge capacity of 246.39 mAh g^−1^ at 0.1 C, far surpassing the pristine NCM811 (82.12% and 194.67 mAh g^−1^). The significantly enhanced coulombic efficiency and specific capacity of the NCM811@3LNO electrode can be attributed to the electronic conductor LaNiO_3_ coating layer that provides the electronic conduction pathway between particles, leading to fast electron transport.

To further investigate the phase transition behavior during the first charge and discharge process, Figure 4b–d presents the corresponding dQ dV^−1^ curves of pristine NCM811, NCM811@LNO, and NCM811@3LNO electrodes, respectively. All the dQ dV^−1^ curves exhibit the phase transitions from hexagonal to monoclinic (H1 to M) and then to other hexagonal (H2 and H3). Clearly, NCM811@LNO (0.0068 V) and NCM811@3LNO (0.0032 V) electrodes demonstrate the lower potential difference of anodic-cathodic peaks compared to that of the pristine NCM811 (0.0096 V). The decreased potential difference of NCM811@3LNO electrode implies improved electrode reversibility and reduced electrochemical polarization according to the literature [40,41]. The above results indicate that surface modification with electronic conductor LaNiO_3_ crystallites is beneficial to improve the electrode kinetics, leading to increasing the first coulombic efficiency and charge/discharge capacity and decreasing the electrochemical polarization degree of NCM811 cathodes.

**Figure 5:**
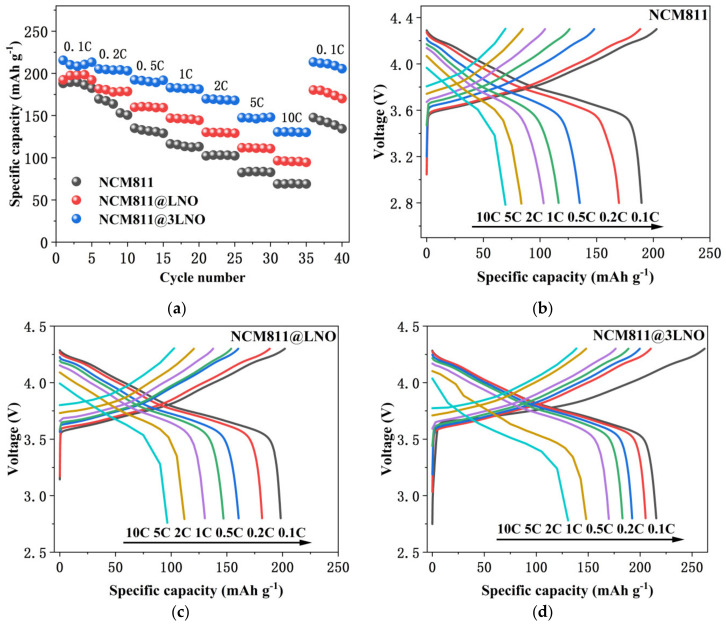
(**a**) Rate performances comparison; charge and discharge profiles of (**b**) pristine NCM811, (**c**) NCM811@LNO, and (**d**) NCM811@3LNO electrodes at different rates.

Figure 5a exhibits the rate capability of pristine NCM811, NCM811@LNO, and NCM811@3LNO electrodes. Additionally, the charge/discharge voltage profiles of all three electrodes at different rates are displayed in Figure 5b–d. Compared with the pristine NCM811, LNO surface-modified NCM811 electrodes exhibit obviously improved high-rate performance. It is conspicuous that a higher discharge capacity of 130.62 mAh g^−1^ is retained after 35 cycles at 10 C for the NCM811@3LNO electrode, corresponding to a capacity loss of 1.12% per cycle from an initial discharge capacity at 0.1 C. In contrast, pristine NCM811 electrode decreases dramatically to 69.50 mAh g^−1^, corresponding to a capacity loss of 1.81% at the same condition. More importantly, NCM811@3LNO exhibits a superior discharge capacity of 213.48 mAh g^−1^ when the current rate recovers back to 0.1 C, far surpassing the pristine NCM811 (147.63 mAh g^−1^). The above results demonstrate that the conductive LaNiO_3_ surface-modified NCM811 cathodes exhibit significantly improved high-rate charge-discharge performance and electrochemical reversibility, suggesting easier electron transport in the LaNiO_3_-modified NCM811.

**Figure 6:**
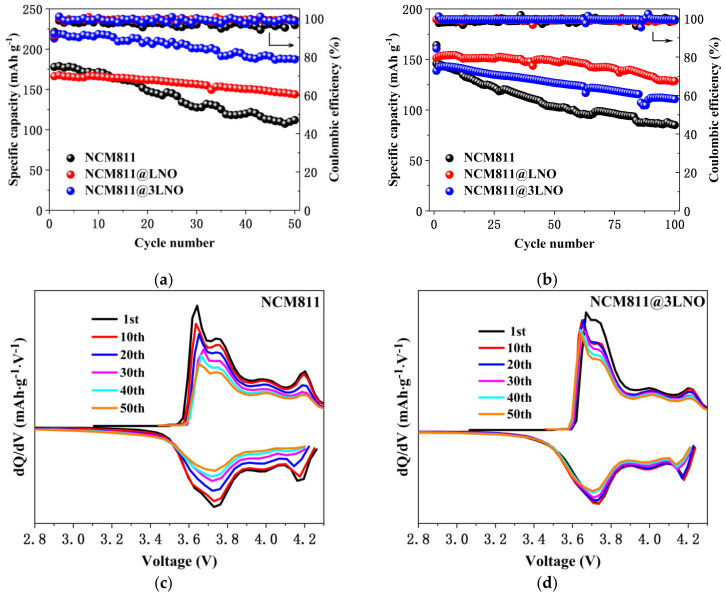
Cycling performances of pristine NCM811, NCM811@LNO, and NCM811@3LNO electrodes at (**a**) 0.5 C and (**b**) 2 C; the corresponding dQ dV^−1^ curves of (**c**) pristine NCM811 and (**d**) NCM811@3LNO electrodes at various cycles at 0.5 C.

The cycling performances of pristine NCM811, NCM811@LNO, and NCM811@3LNO electrodes at 0.5 C are displayed in Figure 6a. At 0.5 C, the NCM811@3LNO electrode exhibits an ultrahigh first discharge capacity of 213.23 mAh g^−1^, significantly surpassing the pristine NCM811 (177.86 mAh g^−1^). Pristine NCM811 exhibits obvious capacity fading with increasing cycle number, maintaining only 62.92% after 50 cycles, and the synchronous decay in the dQ dV^−1^ peaks (Figure 6c) can be observed. In contrast, the NCM811@3LNO electrode shows excellent capacity retention of 87.87% at the same condition, and the corresponding dQ dV^−1^ profiles (Figure 6d) overlap well among various cycles, suggesting outstanding electrochemical reversibility. Besides, Figure 6b shows the cycle performances of all three electrodes at a higher rate of 2 C. Remarkably, the NCM811@LNO electrode maintains 84.95% of its original capacity after 100 cycles, far surpassing 58.31% of the pristine NCM811. These results suggest that the electronic conductor LaNiO_3_ crystallites can provide the electronic conduction pathway between particles, restrain the direct contact between the electrolyte and the cathode surface, thus decreasing the transition metal ions dissolution and the interfacial side reactions, leading to superior cycling stability of the NCM811 cathodes.

**Figure 7:**
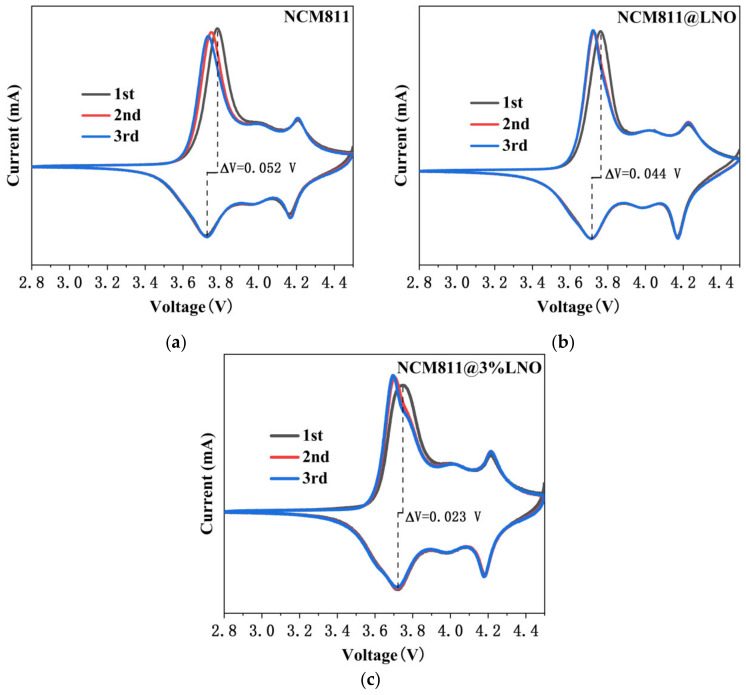
CV curves of (**a**) pristine NCM811, (**b**) NCM811@LNO, and (**c**) NCM811@3LNO electrodes.

To further investigate the redox process and the electrochemical reversibility of NCM811 cathodes during charge-discharge cycling, the first three cyclic voltammograms (Figure 7) were recorded between 2.8 and 4.5 V, and the scan rate was 0.1 mV s^−1^. All electrodes exhibit three redox couples. Compared with pristine NCM811, NCM811@LNO and NCM811@3LNO electrodes exhibit similar CV features, suggesting that the redox process of the NCM811 is not affected by the presence of LaNiO_3_ coating. The potential difference (ΔV) of oxidation-reduction peaks is closely related to the polarization degree of the electrode materials and the reversibility of the electrochemical redox reaction according to the literature [42]. Significantly, the potential difference of the pristine NCM811 electrode is 0.052 V, which surpassed the NCM811@LNO (0.044 V) and NCM811@3LNO (0.023 V) electrodes. Furthermore, compared with pristine NCM811, the CV curves of NCM811@LNO and NCM811@3LNO electrodes overlap well in the 2nd and 3rd cycles, suggesting that the LaNiO_3_-modified LiNi_0.8_Co_0.1_Mn_0.1_O_2_ electrodes have quasi-reversible electrochemical kinetics during the Li^+^ insertion and extraction processes [43]. These results suggest that the electronic conductor LaNiO_3_ coating are conducive to improving the electrochemical reversibility and reducing the polarization degree of the NCM811 cathodes, which are consistent with the results from superior cycling performance and high-rate performance of the NCM811@LNO and NCM811@3LNO electrodes.

**Figure 8:**
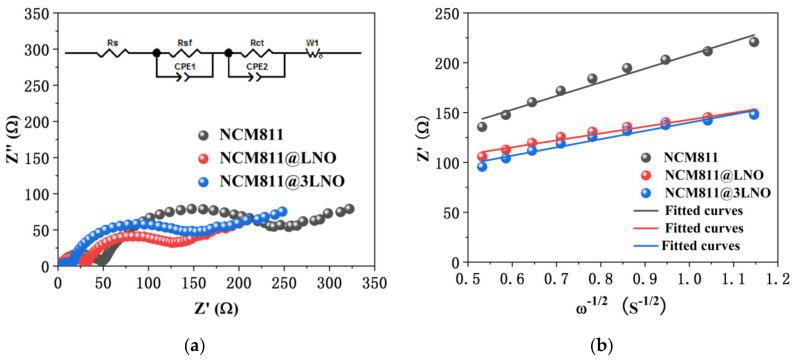
(**a**) Nyquist plots with an equivalent circuit (inset) of pristine NCM811, NCM811@LNO, and NCM811@3LNO electrodes; (**b**) the relationship plots between Z′ and ω^−1/2^ in the low-frequency region.

To study the electrochemical kinetic behaviors for the high-rate performance, EIS of the pristine NCM811, NCM811@LNO, and NCM811@3LNO electrodes were measured after 100 cycles at 2 C. Two semicircles and an inclined line can be seen in all Nyquist plots (Figure 8a). Generally, the solution resistance (R_s_) is represented by the high-frequency intercept at the real axis [44,45]. The surface-film resistance (R_sf_) is represented by the first semicircle at high-frequency, while the charge-transfer resistance (R_ct_) between the electrolyte and cathode materials is represented by the second semicircle at medium-frequency [46]. The inclined line at low-frequency stands for the Warburg impedance (Z_w_), which is related to the lithium ions diffusion in the bulk of cathode materials [47]. Furthermore, the following equation was used to calculate the Li^+^ diffusion coefficient (D_Li_^+^) from the Nyquist plots in the low-frequency region:

(1) $$D_{Li^{{}^{+}}}\;=\;\frac{R^{2}T^{2}}{2A^{2}n^{4}F^{4}C^{2}\sigma^{2}}$$

where C represents the concentration of Li^+^ in the NCM811 cathode, F represents the Faraday constant, A represents the surface area of the electrode, T represents the absolute temperature, *n* represents the amount of the electrons per molecule participating in the electronic transfer reaction, R represents the gas constant, and σ represents the Warburg coefficient, which can be obtained by the following equation from the slope of the linear fitting of resistance (Z′) vs. the reciprocal square roots of the frequency (ω^−1/2^) in the low-frequency region [48]:

(2) $$Z^{\prime }\;=\;R_{s}+R_{\mathrm{ct}}+\sigma \omega^{-1/2}$$

Figure 8b presents the relationship between Z′ and ω^−1/2^. Moreover, Table 3 exhibits the fitting resistance and calculated D_Li_^+^ values. Compared with pristine NCM811, NCM811@LNO and NCM811@3LNO electrodes exhibit lower R_sf_ and R_ct_ values. Additionally, the calculated D_Li_^+^ value for the pristine NCM811 electrode is 1.17 × 10^−14^ cm^2^ s^−1^ after 100 cycles, whereas the D_Li_^+^ value for NCM811@LNO and NCM811@3LNO is 4.61 × 10^−14^ cm^2^ s^−1^ and 3.21 × 10^−14^ cm^2^ s^−1^, respectively. These results are consistent with the results from the cycling performances at 2 C (Figure 6b), indicating that the high conductive LNO coating layer is conducive to facilitating the electron transport, suppressing the side reactions between the electrolyte and NCM811 surface, subsequently improving the electrode kinetics and reducing the interfacial resistance.

## 4. Conclusions

In this paper, electronic conductor LaNiO_3_ crystallite surface-modified LiNi_0.8_Co_0.1_Mn_0.1_O_2_ cathodes were prepared, and the effects of conductive LaNiO_3_ coating on the LiNi_0.8_Co_0.1_Mn_0.1_O_2_ cathodes were studied. The present work indicates that LaNiO_3_ nanoparticles are uniformly distributed on the NCM811 particle surface, which is beneficial towards improving the electrode kinetics caused by the fast electron transport and restraining the direct contact between the electrolyte and cathode materials, leading to excellent cycling performance and high-rate capability of LaNiO_3_-modified LiNi_0.8_Co_0.1_Mn_0.1_O_2_ cathodes. As a result, surface modification with high electronic conductivity oxide is an effective method to improve the electrochemical performances of Ni-rich LiNi_0.8_Co_0.1_Mn_0.1_O_2_ cathodes, which can also be extended to other electrodes for fast charging-discharging LIBs.

## Figures and Tables

**Table 1:** The lattice parameters of all three samples.

Parameter	Pristine NCM811	NCM811@LNO	NCM811@3LNO
a (Å)	2.8731	2.8709	2.8720
c (Å)	14.1963	14.1951	14.1959
c/a	4.9411	4.9444	4.9428
I_(003)_/I_(104)_	1.5893	1.8820	1.6045

**Table 2:** The electrochemical performances of all three electrodes in the initial cycle at 0.1 C.

Electrode	First Charge Capacity (mAh g^−1^)	First Discharge Capacity (mAh g^−1^)	First Coulombic Efficiency (%)
pristine NCM811	237.06	194.67	82.12
NCM811@LNO	218.24	192.28	88.11
NCM811@3LNO	289.53	246.40	85.10

**Table 3:** Impedance parameters of pristine NCM811, NCM811@LNO, and NCM811@3LNO electrodes after fitting.

Electrode	R_s_ (Ω)	R_sf_ (Ω)	R_ct_ (Ω)	D_Li_^+^ (cm^2^ s^−1^)
pristine NCM811	4.35	39.94	173.30	1.17 × 10^−14^
NCM811@LNO	2.88	24.94	74.88	4.61 × 10^−14^
NCM811@3LNO	4.06	8.96	108.50	3.21 × 10^−14^

## Data Availability

The data presented in this study are available from the corresponding authors upon reasonable request.

## References

[1] An J., Shi L.Y., Chen G., Li M., Liu H.J., Yuan S., Chen S., Zhang D. (2017). Insights into the stable layered structure of a Li-rich cathode material for lithium-ion batteries. J. Mater. Chem. A.

[2] Sun Z., Xu L., Dong C., Zhang H., Zhang M., Liu Y., Zhou Y., Han Y., Chen Y. (2019). Enhanced cycling stability of boron-doped lithium-rich layered oxide cathode materials by suppressing transition metal migration. J. Mater. Chem. A.

[3] Park K.J., Lim B.B., Choi M.H., Jung H.G., Sun Y.K., Haro M., Vicente N., Bisquert J., Garcia-Belmonte G. (2015). A high-capacity Li[Ni_0.8_Co_0.06_Mn_0.14_]O_2_ positive electrode with a dual concentration gradient for next-generation lithium-ion batteries. J. Mater. Chem. A.

[4] Braun P.V., Cho J., Pikul J.H., King W.P., Zhang H. (2012). High power rechargeable batteries. Curr. Opin. Solid State Mater. Sci..

[5] Liu Y.Y., Zhu Y.Y., Cui Y. (2019). Challenges and opportunities towards fast-charging battery materials. Nat. Energy.

[6] Wang X., Ding Y.L., Deng Y.P., Chen Z. (2020). Ni-Rich/Co-Poor Layered Cathode for Automotive Li-Ion Batteries: Promises and Challenges. Adv. Energy Mater..

[7] Tomaszewska A., Chu Z.Y., Feng X.N., O’Kane S., Liu X.H., Chen J.Y., Ji C.Z., Endler E., Li R.H., Liu L.S. (2019). Lithium-ion battery fast charging: A review. eTransportation.

[8] Rodrigues M.T.F., Son S.B., Colclasure A.M., Shkrob I.A., Trask S.E., Bloom I.D., Abraham D.P. (2021). How Fast Can a Li-Ion Battery Be Charged? Determination of Limiting Fast Charging Conditions. ACS Appl. Energy Mater..

[9] Kang B., Ceder G. (2009). Battery materials for ultrafast charging and discharging. Nature.

[10] Tang Y.X., Zhang Y.Y., Li W.L., Ma B., Chen X.D. (2015). Rational material design for ultrafast rechargeable lithium-ion batteries. Chem. Soc. Rev..

[11] Yoo G.W., Son J.T. (2016). Improvement of Electrochemical Properties and Thermal Stability of a Ni-rich Cathode Material by Polypropylene Coating. J. Electrochhem. Sci. Technol..

[12] Hu J.W., Fan F.S., Zhang Q., Zhong S.W., Ma Q.X. (2021). Effects of long-term fast charging on a layered cathode for lithium-ion batteries. J. Energy Chem..

[13] Ding X., Li Y.X., Deng M.M., Wang S., Aqsa Y., Hu Q., Chen C.H. (2019). Cesium doping to improve the electrochemical performance of layered Li_1.2_Ni_0.13_Co_0.13_Mn_0.54_O_2_ cathode material. J. Alloys Compd..

[14] Zou P.J., Lin Z.H., Fan M.N., Wang F., Liu Y., Xiong X.H. (2020). Facile and efficient fabrication of Li_3_PO_4_-coated Ni-rich cathode for high-performance lithium-ion battery. Appl. Surf. Sci..

[15] Xin F.X., Zhou H., Chen X.B., Zuba M., Chernova N., Zhou G.W., Whittingham M.S. (2019). Li-Nb-O coating/substitution enhances the electrochemical performance of LiNi_0.8_Mn_0.1_Co_0.1_O_2_ (NMC811) cathode. ACS Appl. Mater. Interfaces.

[16] Becker D., Börner M., Nölle R., Diehl M., Klein S., Rodehorst U., Schmuch R., Winter M., Placke T. (2019). Surface Modification of Ni-rich LiNi_0.8_Co_0.1_Mn_0.1_O_2_ Cathode Material by Tungsten Oxide Coating for Improved Electrochemical Performance in Lithium Ion Batteries. ACS Appl. Mater. Interfaces.

[17] Yang H., Wu H., Ge M., Li L., Yuan Y., Yao Q., Chen J., Xia L., Zheng J., Chen Z. (2019). Simultaneously Dual Modification of Ni-Rich Layered Oxide Cathode for High-Energy Lithium-Ion Batteries. Adv. Funct. Mater..

[18] Hou P., Yin J., Ding M., Huang J., Xu X. (2017). Surface/Interfacial Structure and Chemistry of High-Energy Nickel-Rich Layered Oxide Cathodes: Advances and Perspectives. Small.

[19] Su Y.F., Chen G., Chen L., Li W.K., Zhang Q.Y., Yang Z.R., Lu Y., Bao L.Y., Tan J., Chen R.J. (2018). Exposing the {010} Planes by Oriented Self-Assembly with Nanosheets To Improve the Electrochemical Performances of Ni-Rich Li[Ni_0.8_Co_0.1_Mn_0.1_]O_2_ Microspheres. ACS Appl. Mater. Interfaces.

[20] Chen Z., Nguyen H., Zarrabeitia M., Liang H., Geiger D., Kim J., Kaiser U., Passerini S., Iojoiu C., Bresser D. (2021). Lithium Phosphonate Functionalized Polymer Coating for High-energy Li[Ni_0.8_Co_0.1_Mn_0.1_]O_2_ with Superior Performance at Ambient and Elevated Temperatures. Adv. Funct. Mater..

[21] Jamil S., Wang G., Yang L., Xie X., Cao S., Liu H., Chang B., Wang X. (2020). Suppressing H2–H3 phase transition in high Ni–low Co layered oxide cathode material by dual modification. J. Mater. Chem. A.

[22] Liu Y., Tang L.B., Wei H.X., Zhang X.H., He Z.J., Li Y.J., Zheng J.C. (2019). Enhancement on structural stability of Ni-rich cathode materials by in-situ fabricating dual-modified layer for lithium-ion batteries. Nano Energy.

[23] Lee S., Kim M., Jeong J., Kim D., Chung K., Roh K., Kim K. (2017). Li_3_PO_4_ surface coating on Ni-rich LiNi_0.6_Co_0.2_Mn_0.2_O_2_ by a citric acid assisted sol-gel method: Improved thermal stability and high-voltage performance. J. Power Sources.

[24] Song B.H., Li W.D., Oh S.M., Manthiram A. (2017). Long-Life Nickel-Rich Layered Oxide Cathodes with a Uniform Li_2_ZrO_3_ Surface Coating for Lithium-Ion Batteries. ACS Appl. Mater. Interfaces.

[25] Ran Q.W., Zhao H.Y., Hu Y.Z., Shen Q.Q., Liu W., Liu J.T., Shu X.H., Zhang M.L., Liu S.S., Tan M. (2018). Enhanced electrochemical performance of dual-conductive layers coated Ni-rich LiNi_0.6_Co_0.2_Mn_0.2_O_2_ cathode for Li-ion batteries at high cut-off voltage. Electrochim. Acta.

[26] Li D., Xie R., Tian M., Ma S., Gou L., Fan X., Shi Y., Yong H., Hao L. (2014). Improving high-rate performance of mesoporous Li_2_FeSiO_4_/Fe_7_SiO_10_/C nanocomposite cathode with a mixed valence Fe_7_SiO_10_ nanocrystal. J. Mater. Chem. A.

[27] Sim S.J., Lee S.H., Jin B.S., Kim H.S. (2020). Use of carbon coating on LiNi_0.8_Co_0.1_Mn_0.1_O_2_ cathode material for enhanced performances of lithium-ion batteries. Sci. Rep..

[28] Hu Z.G., Li W.W., Li Y.W., Zhu M., Zhu Z.Q., Chu J.H. (2009). Electronic properties of nanocrystalline LaNiO_3_ and La_0.5_Sr_0.5_CoO_3_ conductive films grown on silicon substrates determined by infrared to ultraviolet reflectance spectra. Appl. Phys. Lett..

[29] Zhang X.D., Hao J.J., Wu L.C., Guo Z.M., Ji Z.H., Luo J., Chen C.G., Shu J.F., Long H.M., Yang F. (2018). Enhanced electrochemical performance of perovskite LaNiO_3_ coating on Li_1.2_Mn_0.54_Ni_0.13_Co_0.13_O_2_ as cathode materials for Li-ion batteries. Electrochim. Acta.

[30] Hofer H.E., Schmidberger R. (1994). Electronic Conductivity in the La(Cr, Ni)O_3_ Perovskite System. J. Electrochem. Soc..

[31] Chen M.S., Wu T.B., Wu J.M. (1996). Effect of textured LaNiO_3_ electrode on the fatigue improvement of Pb(Zr_0.53_Ti_0.47_)O_3_ thin films. Appl. Phys. Lett..

[32] Rajeev K.P., Shivashankar G.V., Raychaudhuri A.K. (1991). Low-Temperature Electronic Properties of a Normal Conducting Perovskite Oxide (LaNiO_3_). Solid State Commun..

[33] Fowlie J., Gibert M., Tieri G., Gloter A., Iniguez J., Filippetti A., Catalano S., Gariglio S., Schober A., Guennou M. (2017). Conductivity and Local Structure of LaNiO_3_ Thin Films. Adv. Mater..

[34] Babulal L.M., Yang C.C., Wu S.H., Chien W.C., Lue S.J. (2020). Enhanced performance of a Ni-rich LiNi_0.8_Co_0.1_Mn_0.1_O_2_ cathode material formed through Taylor flow synthesis and surface modification with Li_2_MoO_4_. Chem. Eng. J..

[35] Li S.M., Wu J., Li J.Y., Liu G.B., Liu H. (2021). Facilitated Coating of Li_3_PO_4_ on the Rough Surface of LiNi_0.85_Co_0.1_Mn_0.05_O_2_ Cathodes by Synchronous Lithiation. ACS Appl. Energy Mater..

[36] Ryu H.H., Park G.T., Chong S.Y., Sun Y.K. (2019). Suppressing detrimental phase transitions via tungsten doping of LiNiO_2_ cathode for next-generation lithium-ion batteries. J. Mater. Chem. A.

[37] Si Z., Shi B.Z., Huang J., Yu Y., Han Y., Zhang J.L., Li W. (2021). Titanium and fluorine synergetic modification improves the electrochemical performance of Li(Ni_0.8_Co_0.1_Mn_0.1_)O_2_. J. Mater. Chem. A.

[38] Zheng J.X., Ye Y.K., Liu T.C., Xiao Y.G., Wang C.M., Wang F., Pan F. (2019). Ni/Li Disordering in Layered Transition Metal Oxide: Electrochemical Impact, Origin, and Control. Acc. Chem. Res..

[39] Retuerto M., Pereira A.G., Pérez-Alonso F.J., PenA M.A., Fierro J.L., Alonso J.A., Fernández-Díaz M.T., Pascual L., Rojas S. (2017). Structural effects of LaNiO_3_ as electrocatalyst for the oxygen reduction reaction. Appli. Catal. B Environ..

[40] Ding G.Y., Li Y.H., Gao Y., Wang Q.L., Zhu Z., Jing X.G., Yan F.Q., Yue Z.H., Li X.M., Sun F.G. (2020). Uniform Coating of Se on Selenophilic Surfaces of Nickel-Rich Layered Oxide Cathode Materials for High Performance Li-Ion Batteries. ACS Sustain. Chem. Eng..

[41] Huang Y.P., Yao X., Hu X.C., Han Q.Y., Wang S.Q., Ding L.X., Wang H.H. (2019). Surface coating with Li-Ti-O to improve the electrochemical performance of Ni-rich cathode material. Appl. Surf. Sci..

[42] Zhang D.K., Liu Y., Wu L., Feng L.W., Jin M.L., Zhang R., Jin M.L. (2019). Effect of Ti ion doping on electrochemical performance of Ni-rich LiNi_0.8_Co_0.1_Mn_0.1_O_2_ cathode material. Electrochim. Acta.

[43] Mofid W.E., Ivanov S., Konkin A., Bund A. (2014). A high performance layered transition metal oxide cathode material obtained by simultaneous aluminum and iron cationic substitution. J. Power Sources.

[44] Song X., Liu G.X., Yue H.F., Luo L., Yang S.Y., Huang Y.Y., Wang C.R. (2020). A Novel Low-Cobalt Long-Life LiNi_0.88_Co_0.06_Mn_0.03_Al_0.03_O_2_ Cathode Material for Lithium Ion Batteries. Chem. Eng. J..

[45] Yuan J., Wen J.W., Zhang J.B., Chen D.M., Zhang D.W. (2017). Influence of calcination atmosphere on structure and electrochemical behavior of LiNi_0.6_Co_0.2_Mn_0.2_O_2_ cathode material for lithium-ion batteries. Electrochim. Acta.

[46] Yang Z.G., Xiang W., Wu Z.G., He F.R., Zhang J., Xiao Y., Zhong B.H., Guo X.D. (2017). Effect of niobium doping on the structure and electrochemical performance of LiNi_0.5_Co_0.2_Mn_0.3_O_2_ cathode materials for lithium ion batteries. Ceram. Int..

[47] Zhang L.J., Jiang J.C., Zhang C.P., Wu B.R., Wu F. (2016). High-rate layered lithium-rich cathode nanomaterials for lithium-ion batteries synthesized with the assist of carbon spheres templates. J. Power Sources.

[48] Meng J.X., Xu H.Z., Ma Q.X., Li Z.F., Xu L.S., Chen Z.J., Cheng B.M., Zhong S.W. (2019). Precursor pre-oxidation enables highly exposed plane {010} for high-rate Li-rich layered oxide cathode materials. Electrochim. Acta.

